# Calf Compression Sleeves Change Biomechanics but Not Performance and Physiological Responses in Trail Running

**DOI:** 10.3389/fphys.2017.00247

**Published:** 2017-04-27

**Authors:** Hugo A. Kerhervé, Pierre Samozino, Fabrice Descombe, Matthieu Pinay, Guillaume Y. Millet, Marion Pasqualini, Thomas Rupp

**Affiliations:** ^1^Laboratoire Interuniversitaire de Biologie de la Motricité, EA 7424, Université Savoie Mont BlancChambéry, France; ^2^Faculty of Science, Health, Education and Engineering, School of Health and Sport Sciences, University of the Sunshine CoastSippy Downs, QLD, Australia; ^3^Medipôle de SavoieChalles-les-Eaux, France; ^4^Human Performance Laboratory, Faculty of Kinesiology, University of CalgaryCalgary, Canada; ^5^ThuasneSt-Etienne, France

**Keywords:** performance, fatigue, running biomechanics, muscle oxygenation, prolonged exercise, leg stiffness

## Abstract

**Introduction:** The aim of this study was to determine whether calf compression sleeves (CS) affects physiological and biomechanical parameters, exercise performance, and perceived sensations of muscle fatigue, pain and soreness during prolonged (~2 h 30 min) outdoor trail running.

**Methods:** Fourteen healthy trained males took part in a randomized, cross-over study consisting in two identical 24-km trail running sessions (each including one bout of running at constant rate on moderately flat terrain, and one period of all-out running on hilly terrain) wearing either degressive CS (23 ± 2 mmHg) or control sleeves (CON, <4 mmHg). Running time, heart rate and muscle oxygenation of the medial *gastrocnemius* muscle (measured using portable near-infrared spectroscopy) were monitored continuously. Muscle functional capabilities (power, stiffness) were determined using 20 s of maximal hopping before and after both sessions. Running biomechanics (kinematics, vertical and leg stiffness) were determined at 12 km·h^−1^ at the beginning, during, and at the end of both sessions. Exercise-induced Achilles tendon pain and delayed onset calf muscles soreness (DOMS) were assessed using visual analog scales.

**Results:** Muscle oxygenation increased significantly in CS compared to CON at baseline and immediately after exercise (*p* < 0.05), without any difference in deoxygenation kinetics during the run, and without any significant change in run times. Wearing CS was associated with (i) higher aerial time and leg stiffness in running at constant rate, (ii) with lower ground contact time, higher leg stiffness, and higher vertical stiffness in all-out running, and (iii) with lower ground contact time in hopping. Significant DOMS were induced in both CS and CON (>6 on a 10-cm scale) with no difference between conditions. However, Achilles tendon pain was significantly lower after the trial in CS than CON (*p* < 0.05).

**Discussion:** Calf compression did not modify muscle oxygenation during ~2 h 30 of trail running but significantly changed running biomechanics and lower limb muscle functional capabilities toward a more dynamic behavior compared to control session. However, wearing compression sleeves did not affect performance and exercise-induced DOMS, while it minimized Achilles tendon pain immediately after running.

## Introduction

Compression garments are widely used in the treatment or prevention of clinical, occupational, and travel-related ailments for their beneficial effect on venous hemodynamics. The extrinsic mechanical pressure they provide to the underlying soft tissues increases cutaneous and subcutaneous interstitial pressure, thereby reducing peripheral blood pooling, leg swelling, and improving venous return (Partsch et al., 2008). Compression garments are also used in healthy populations for post-exercise recovery purposes, although there is no definitive consensus about their effects (MacRae et al., 2011; Born et al., 2013; Hill et al., 2014). Overall, it has been largely demonstrated that compression garments improve perfusion and increase local muscle oxygenation at rest (Bochmann et al., 2005; Bringard et al., 2006), so that many of its expected ergogenic effects are dependent on the subsequent compensation of local oxygen deficit contracted during exercise and fastening of energy stocks reconstitution (Di Prampero et al., 1983).

During the last decade, wearing compression garments during exercise has also become increasingly popular in sports such as running and cycling. Similar to the literature specific to post-exercise recovery, various outcomes have been reported, with no or small beneficial effects in physiological, psychological, or biomechanical parameters (Engel et al., 2016). Wearing compression garments during running exercise was associated with improvements in muscle oxygenation during intermittent high intensity running (Sear et al., 2010), leg volume (Bovenschen et al., 2013), muscle damage using magnetic resonance imaging and histochemical techniques (Valle et al., 2013), delayed onset of muscle soreness (DOMS, see Duffield and Portus, 2007), and heart rate (Varela-Sanz et al., 2011). Positive effects in performance have also been noted in incremental tests (Kemmler et al., 2009; Sear et al., 2010), repeated sprinting (Higgins et al., 2009; Born et al., 2014), and jumping height following submaximal exercise (Rugg and Sternlicht, 2013; Bieuzen et al., 2014) or after a 10 km run (Ali et al., 2011). Conversely, other studies have reported no measurable effect on limb volume (Areces et al., 2015), fractional oxygen utilization (Kemmler et al., 2009; Wahl et al., 2011; Born et al., 2014; Priego Quesada et al., 2015; Stickford et al., 2015), muscle oxygenation or blood flow (Vercruyssen et al., 2012; Born et al., 2014), heart rate or indicators of central cardiovascular adaptations (Ali et al., 2007; Sperlich et al., 2011; Wahl et al., 2011; Vercruyssen et al., 2012; Born et al., 2014; Priego Quesada et al., 2015), lactate or exercise metabolite removal (Kemmler et al., 2009; Ali et al., 2010; Sperlich et al., 2011; Wahl et al., 2011; Vercruyssen et al., 2012; Areces et al., 2015), ratings of perceived exertion (RPE) or DOMS (Ali et al., 2007, 2010; Bovenschen et al., 2013; Areces et al., 2015; Priego Quesada et al., 2015), running economy and gait kinematics (Varela-Sanz et al., 2011; Stickford et al., 2015; Vercruyssen et al., 2016), maximal voluntary and evoked contractions (Vercruyssen et al., 2016), as well as performance in repeated sprinting (Duffield et al., 2008), or in running performed at maximal (Ali et al., 2007; Priego Quesada et al., 2015) and at sub-maximal exercise intensities (Ali et al., 2007, 2011; Wahl et al., 2011; Vercruyssen et al., 2012; Priego Quesada et al., 2015). This abundant but heterogeneous literature may underline probable task-dependent ergogenic effects of the compression when used during exercise.

Recent studies have indicated that the mechanical support provided by compression garments may contribute to reduce the transmission of oscillations or vibrations (Doan et al., 2003; MacRae et al., 2011; Bovenschen et al., 2013), which may in turn reduce fatigue (Miyamoto et al., 2011), and increase performance (Kraemer et al., 1998). Because minimizing musculo-skeletal damage and fatigue is considered paramount for performance in trail running (Millet, 2011), and since prolonged running on trails has been shown to alter footstrike patterns and gait biomechanics (Morin et al., 2011b; Vernillo et al., 2014; Giandolini et al., 2016), it is possible that compression garments have beneficial effects in situations maximizing the exposure to fatigue, shocks and vibrations, such as prolonged running exercise performed on trails with pronounced elevation gain and loss. Despite the increased popularity of trail running over short and long distances, there is only limited evidence available on the effects of compression garments worn during running on trails with uphill and downhill sections (Vercruyssen et al., 2012, 2016; Bieuzen et al., 2014), or for road running for durations longer than ~90 min (Areces et al., 2015). These studies indicate that while compression garments worn during trail running may reduce muscle soreness post-run and the recovery of lower limb power capacity (Bieuzen et al., 2014), there has currently been no evidence of the ergogenic effect of compression garments on the running pace and performance (Vercruyssen et al., 2012; Bieuzen et al., 2014; Vercruyssen et al., 2016). However, all previously cited studies have performed measurements before and after running, and no study on prolonged running >90 min has been designed to measure physiological and biomechanical adaptations of running with compression garments during prolonged trail running.

Therefore, the aim of this study was to determine if wearing calf compression sleeves (CS, compression garments covering the lower limb between the ankle and knee joints) during a prolonged running exercise (~150 min) performed on trails with marked elevation gain and loss, had a measurable effect on local muscle tissue oxygenation, running pattern, muscle power capability, performance, and subjective perception of muscle fatigue, pain and soreness. Because of the various changes in contraction modes and intensities characteristic of trail running with marked elevation gain and loss (changes in gradient, directions, and surfaces), we hypothesized that wearing CS may benefit from improved perfusion and local muscle oxygenation. We also hypothesized that wearing CS would reduce the deleterious effects of fatigue on running biomechanics, muscle power capability and subjective perception, thereby improving performance.

## Methods

### Ethics statement and participants

This study, including all the procedures described has been explicitly approved by national ethics committees (Comité de Protection des Personnes, Ref. IDRCB-2014-A01721-46 and Agence Nationale de Sécurité du Médicament et des produits de santé, Ref. 41504B-81). Participants for this study were recruited in the local running community (clubs, online forums and websites). The inclusion criteria for this study were: male, training in running >2 h weekly, experienced in trail running, successfully performing screening tests, receiving medical insurance, not participating in another clinical study, and not planning to participate in sporting competition, unusual or >90 min exercises during the study period.

All participants meeting the inclusion criteria received an information sheet describing the study procedures in detail, and were invited for a ~30 min screening session under the supervision of the study physician. Clearance for participation in the study was granted by the physician, and conditional on providing written informed consent and presenting a normal resting electro-cardiogram. A total of 14 male participants were included in the study (age: 21.7 ± 3.0 year; height: 180.2 ± 4.7 cm; weight: 72.3 ± 6.7 kg; Body Mass Index: 22.2 ± 1.6 kg·m^2^; weekly physical activity volume: 6.00 ± 2.02 h).

### Procedures

Participants in the study completed two experimental sessions, separated by 27 ± 6 days in order to prevent from potential carry-over and fatigue effects between conditions. For normalization purposes, participants (i) were instructed to wear the same clothing and shoes for both sessions, (ii) performed the two sessions at the same time of day (±2 h) and in similar temperature and weather conditions, and (iii) ingested the same volume of standard isotonic drink on both runs (determined from the volume ingested during the first session) carried using a hydration belt during the two sessions (19 g·L^−1^: 61% saccharose, 17% dextrose, 15% maltodextrin).

After a normalized warm-up (light intensity running lasting 10 min, one set of technical drills, and 20 s of hopping), each session consisted in performing a ~24 km running exercise, wearing in a randomized order full tights exerting no compression (CON; Kalenji, Decathlon, France) or degressive calf compression sleeves (CS; UP, Thuasne Sport, Levallois-Perret, France) and ¾ non compressive tights (Kalenji, Decathlon, France), the latter aiming at minimizing the thermoregulatory and proprioceptive differences between conditions. In the current study, calf compression sleeves were preferred over other types of garments since these are a popular choice allowing trail runners to select socks according to individual preferences, which is an important parameter in prolonged running. The actual compression exerted by the garments in the CON (<4 mmHg) and CS (23 ± 2 mmHg) conditions were measured at the beginning of each session via a pressure transducer (PicoPress, Microlab Elettronica, Nicolò, Italy) placed between the medial and lateral heads of the muscle *gastrocnemius* with the participants standing in a relaxed, balanced position.

**Figure 1:**
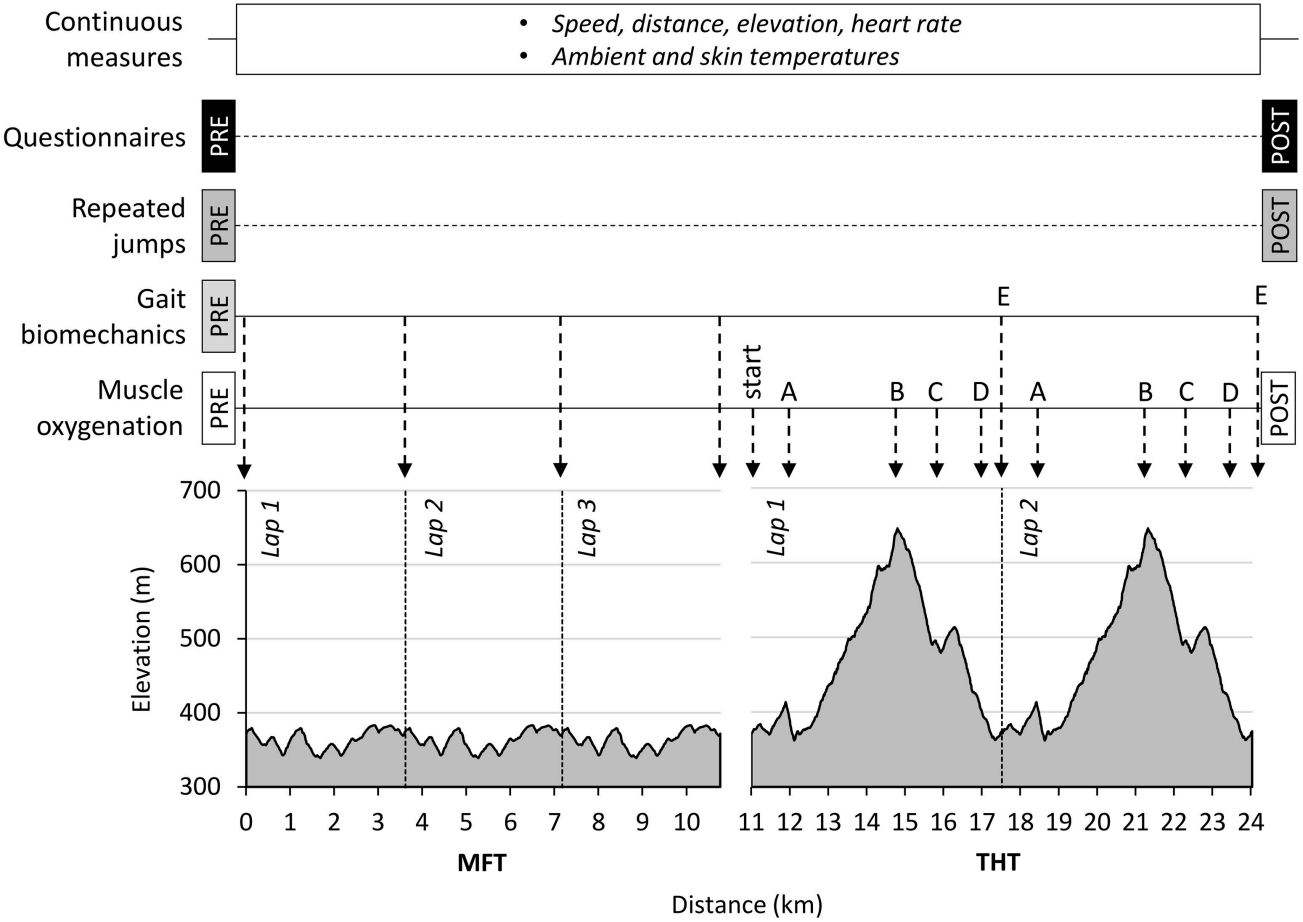
**Experimental protocol**. Schematic representation of the protocol, measures performed, and elevation gain and loss as a function of distance in the running exercise.

The two sessions were performed on the same signposted ~24 km course (total elevation change [D±] of 1,020 m, 90% trail; see Figure 1), consisting of one period performed at constant rate on moderately flat terrain (MFT, three laps of the same 3.6 km course, D±: 90 m) and one period performed all-out on a technical and hilly terrain (THT, two laps of the same 6.6 km course, D±: 375 m), both separated by approximately 10 min for muscle oxygenation measurements. All participants were local to the testing venue, were familiarized with the entire course several times before testing, and ran alone during each session to minimize the effect of group pacing, but were provided with timing feedback from investigators and carried a watch to pace their effort accordingly throughout MFT.

Heart rate (HR), speed and time (via Global Positioning System, GPS) were recorded continuously at 0.2 Hz using a wristwatch and chest strap (ForeRunner 405, Garmin, Olathe, KS; GPS precision: 2.5 m at 1 Hz; GPS tracking sensitivity: −143 dBm). A 0.2 Hz sampling frequency was used to maximize battery life and has previously been used to monitor the speed of trail runners (Kerhervé et al., 2015). The course elevation profile was recalculated using an online mapping utility (www.tracedetrail.fr). Each participant's HR was expressed as a percentage of theoretical maximum HR (%HR) using Equation (1) (Tanaka et al., 2001):

(1) % HR=208−0.7×(age  in  years)

In order to minimize the effect of ambient temperature on the measures, testing was performed under similar meteorological conditions. Ambient temperature and skin temperature under the garments were measured at 0.017 Hz using two wireless temperature sensors (Ibutton-Thermocron, Maxim Integrated, San Jose, CA), one secured to the hydration belt, and one affixed directly to the skin on the calf muscle *gastrocnemius medialis*, respectively.

#### Muscle oxygenation

To investigate muscle oxygenation, the portable near-infrared spectroscopy (NIRS) apparatus used in this study was a 2-wavelength continuous wave system, allowing to assess changes in oxy- (Δ[O_2_Hb]), deoxy- (Δ[HHb]) and total hemoglobin (Δ[tHb]) concentrations relative to an arbitrary baseline, in the investigated muscle area. The device simultaneously uses the modified Beer-Lambert law and spatially resolved spectroscopy method to measure hemoglobin changes from the differences in absorption characteristics of the light (750 and 850 nm) and to compute a tissue saturation index (TSI, %), which reflects the average saturation of the underlying muscle tissue. Given the uncertainty of the proton pathlength at rest and during exercise, we used an arbitrary value for the differential pathlength of 4.16 based on previous literature (Duncan et al., 1996). The probe was affixed to the skin of the calf muscle *gastrocnemius medialis* of the right leg using double-sided tape, and secured with adhesive bandages. The probe was fixed on the muscle belly parallel to muscle fibers, in a position normalized across conditions and participants at 8.8 ± 1.7 cm under the popliteal fossa. A surgical marker was used to mark the probe placement for accurate repositioning. Skinfold thickness at the site of application of the NIRS probe was determined using Harpenden skinfold calipers (British Indicators Ltd, Burgess Hill, UK). The calculated value of skin and subcutaneous tissue thickness was less than half the mean distance between the sources and the detector (i.e., 35 mm). For subsequent analysis of the TSI, the NIRS signal was smoothed using a Gaussian moving average with a 3-s period. We reported the measures of TSI as the average of the 20 s before each of the following time points: before and after the warm-up in a seated position, at the beginning and at the end of the MFT laps, at the beginning and five times during (points A, B, C, and D corresponding to marked changes in elevation gain and loss, see Figure 1) and at the end of the THT laps, and at the end of the test in a quiet, standing position.

Other indicators of local tissue oxygenation levels such as muscle tissue perfusion (mBF) and muscle tissue oxygen consumption ($\text{m}\dot{\text{V}}{\text{O}}_{2}$) were obtained using venous and arterial occlusions before (PRE), between the MFT and THT, and after the test (POST). A standard pneumatic occlusion cuff (Spengler, Antony, France) was positioned on the right leg of the participants in a standing, balanced position, and with their weight distributed slightly more to the left leg. The mBF (in mL·min·100mL^−1^) was estimated from the following equation (Van Beekvelt, 2002):

(2) mBF=([Δ[tHb]×60([Hb]×1,000)/4 ]×1,000)÷10

where the slope of [tHb] (expressed in μM·s^−1^) was measured by NIRS during two 20-s venous occlusions (70 mmHg) separated by 45 s of rest, and [Hb] is the absolute value of hemoglobin concentration (group mean: 9.56 ± 0.67 mmol×L^−1^) assessed for each subject from a micro-sample of blood at the finger with an hemoglobin photometric analyzer (Hemo Control, EKF Diagnostics, UK).

As previously described (Hamaoka et al., 2007), $\text{m}\dot{\text{V}}{\text{O}}_{2}$ was estimated using the initial rate of muscle deoxygenation measured by NIRS during two 20-s arterial occlusions (280 mmHg) separated by 45 s of rest. Assuming a value of 1.04 kg·L^−1^ for muscle density and that during the occlusion [tHb] remains roughly constant, the linear rate of increase in [HHb] or the linear rate of decrease in [O_2_Hb] (expressed in μM×s^−1^) was converted to milliliters O_2_ per minute per 100 g tissue (in mL×min×100 g^−1^) using the following equation (Van Beekvelt, 2002):

(3) mV˙O2=abs([Δ[O2Hb]×6010×1.04] ×4)× 22.41,000

#### Muscle functional capabilities

Muscle functional capabilities of the lower limbs were assessed during a bout of 20 s of maximal hopping (repeated jumps) before (rested state, PRE) and after (fatigued state, POST) the running exercise (Joseph et al., 2013). Participants were asked to jump as high and as often as possible over the whole 20 s bout. Contact time (*t*_*c*_), aerial time (*t*_*a*_) and jump frequency (f = 1/[*t*_*a*_ + *t*_*c*_]) were measured using optoelectric cells (OptoJumpNext, Microgate, Bolzano, Italy) positioned ~1-m apart on level ground, with a time resolution of 1 ms. In order to maximize the contribution of plantar flexor muscles and minimize the contribution of knee extensor muscles, the jumps were performed without bending the knees using verbal instructions and a knee brace locked in a fully extended position (GenuControl, Thuasne Sport). Average and maximum power (in W·kg^−1^) and limb stiffness (*kl*_*eg*_, in N·m^−1^) were calculated based on previous validated computations using *t*_*c*_ and *t*_*a*_ (Bosco et al., 1983; Dalleau et al., 2004), following equations 4, 5, and 6:

(4) h=g×ta28

(5) P=(g2×ta×[ta+tc])4×tc

(6) kleg=(m×Π×[ta+tc])tc2×(ta+tcΠ−tc4)

where g is the gravitational attraction (g = 9.81 m·s^−1^), m is the mass of the participant (in kg).

#### Biomechanical running pattern

Running pattern was evaluated through determination of contact time (*t*_*c*_), aerial time (*t*_*a*_), stride frequency (f = 1/[*t*_*a*_ + *t*_*c*_]), duty factor (DF), lower limb (*k*_*leg*_) and vertical stiffness (*k*_*vert*_) once before (PRE), and 5 times during each running test (at the end of each lap in MFT and THT) using a series of optoelectric cells (OptoJumpNext, Microgate, Bolzano, Italy) over an 8-m length. Each time, the participants were required to run between the optoelectric cells at an imposed speed of ~12 km·h^−1^, paced using a cyclist and a 100-m run-up (Morin et al., 2011b). The percentage of ground contact time during each stride, i.e., duty factor (DF, expressed as a percentage), was subsequently calculated as DF = [*t*_*c*_/*t*_*c*_ + *t*_*a*_] × 100. Lower limb stiffness (*k*_*leg*_, in N·m^−1^) and vertical stiffness (*k*_*vert*_, in N·m^−1^) were calculated for each trial using Equations (7.1–7.5):

(7.1) $$k_{leg}=F_{max}/△\text{L}$$

(7.2) $$△\text{L}=\text{L}-\sqrt{{\text{L}}^{2}-\left(\text{v}t_{\text{c}}-\text{d}/2\right)}+△\text{z}$$

(7.3) $$k_{vert}=F_{max}/△\text{z}$$

(7.4) $$F_{max}=\text{m}\times \text{g}\times \left(\pi /2\right)\times \left(t_{a}/t_{c}+1\right)$$

(7.5) $$△\text{z}=\left(F_{max}/\text{m}\right)\times \left({t_{c}}^{2}/\pi^{2}\right)+\left(\text{g }\cdot \;{t_{c}}^{2}/8\right)$$

where m is the mass of the participant (in kg), L is the length of the lower limb (in m), Δz (in m) is the vertical variation of the center of mass, ΔL (in m) is the variation of leg length during the stance phase, and *F*_*max*_ is the vertical component of peak force during the stance phase. A correction of L (d = 0.18×L) was used to take into account the foot landing characteristics (Bullimore and Burn, 2006).

#### Questionnaires

Five separate linear visual analog scales were used to measure subjective ratings of the sensations of fatigue (Lee et al., 1991) and discomfort/pain (Hawker et al., 2011) in the calf and thigh muscles, and Achilles' tendon PRE and immediately POST running exercise. The delayed onset of muscle soreness (DOMS) at the calf muscle level was measured using the same linear visual analog scale 1, 24, 48, 72 h, and 7 days after the running exercise. Visual analog scales were ranked from 0 (no fatigue/discomfort/pain) to 10 (worst fatigue/discomfort/pain imaginable), and participants could choose either full or in-between increments to grade the sensation.

#### Variables and statistical analyses

In order to ensure the CON and CS conditions were performed in a comparable environmental and physical state, we reported ten standardization parameters across conditions: participants weight, average ambient and skin temperatures over the two sessions, localized sensations of fatigue and pain before the beginning of exercise, performance during MFT (total time), and time stopped between the MFT and THT. In order to evaluate the differences between conditions at rest, we compared the baseline measures of mBF and $\text{m}\dot{\text{V}}{\text{O}}_{2}$ with and without CS, and the measures of TSI before and after the warm-up.

All pairs of data in the two conditions (CON and CS) were initially tested for normality (Kolmogorov-Smirnov test) and homogeneity of variances (Fisher's test). We used paired Student's *t*-tests to measures potential significant differences in two means across conditions (CON vs. CS), and repeated-measures ANOVA (two way: condition × time point, three way: condition × lap × time point) with Fisher's LSD *post-hoc* tests when there were comparisons across more than two levels. In cases where the assumptions of normality and homogeneity of variances were not met, the non-parametric Wilcoxon test signed-rank test was used for the comparisons of two means. For pairwise comparisons, we reported effect size using Cohen's *d*, calculated in the standard manner (*d* = $\left({\bar{X}}_{1}\;-\;{\bar{X}}_{2}\right)\;/\;\sigma_{pooled}$, where $\sigma_{pooled}=\sqrt{\left(\left[\sigma_{1}+\sigma_{2}\right]/2\right)}$) interpreted according to Cohen's scale (small effect: 0.2 < *d* < 0.5, medium effect: 0.5 < *d* < 0.8, and large effect: *d* > 0.8). For ANOVAs, we reported effect size using partial eta-squared (η^2^_*p*_) interpreted according to Cohen's scale (small effect: 0.01 < η^2^_*p*_ < 0.06, medium effect: 0.06 < η^2^_*p*_ < 0.14, and large effect: η^2^_*p*_ > 0.14).

All statistical analyses were performed using Statistica (version 13, StatSoft, Inc., Tulsa, OK, USA). The level of significance was set at *p* < 0.05 and data are presented as Mean ± SD, unless stated otherwise.

## Results

All 14 participants successfully completed the two sessions. There were no significant differences between conditions (CON vs. CS) in standardization parameters weight, ambient and skin temperatures, in the subjective sensations of fatigue or pain prior to running, in the time to complete the small laps, and in the recovery durations between MFT and THT (Table 1).

**Table 1:** **Standardization parameters**.

	***n***	**CON**	**CS**	**−95% CI**	**+95% CI**	***p***	***d***
Weight (kg)	14	72.4 ± 6.8	72.1 ± 6.82	−0.09	0.56	0.14	−0.03
Ambient temperature (°C)	14	18.2 ± 4.8	17.9 ± 3.86	−2.52	3.12	0.82	−0.07
Skin temperature (°C)	14	30.0 ± 2.2	29.4 ± 2.10	−0.55	1.86	0.26	−0.28
Fatigue: calf muscle	14	0.79 ± 1.12	1.00 ± 0.96	−0.94	0.51	0.53	0.20
Fatigue: thigh muscle	14	1.29 ± 0.99	0.86 ± 0.86	−0.11	0.97	0.11	−0.46
Pain: calf muscle	14	0.79 ± 1.19	0.79 ± 0.89	−0.82	0.82	1.00	0.00
Pain: thigh muscle	14	0.57 ± 0.76	0.64 ± 0.84	−0.49	0.35	0.72	0.09
Pain: Achilles' tendon	14	0.21 ± 0.58	0.21 ± 0.80	−0.23	0.23	1.00	0.00
Time for MFT (min)	14	60.0 ± 6.5	59.9 ± 6.11	−0.85	0.53	0.62	−0.03
Testing time (min)	14	9.45 ± 1.05	10.1 ± 2.59	−0.79	2.03	0.36	0.31

*Pairwise comparisons (95% CI of the difference, p-value of t-test and Cohen's d effect size) for the average ambient and skin temperatures, the sensations of fatigue and pain at the calf, thigh and Achilles' tendon sites before the run, and total run time during MFT, in control (CON) and compression sleeves (CS) conditions*.

*Values are mean ± SD*.

**Figure 2:**
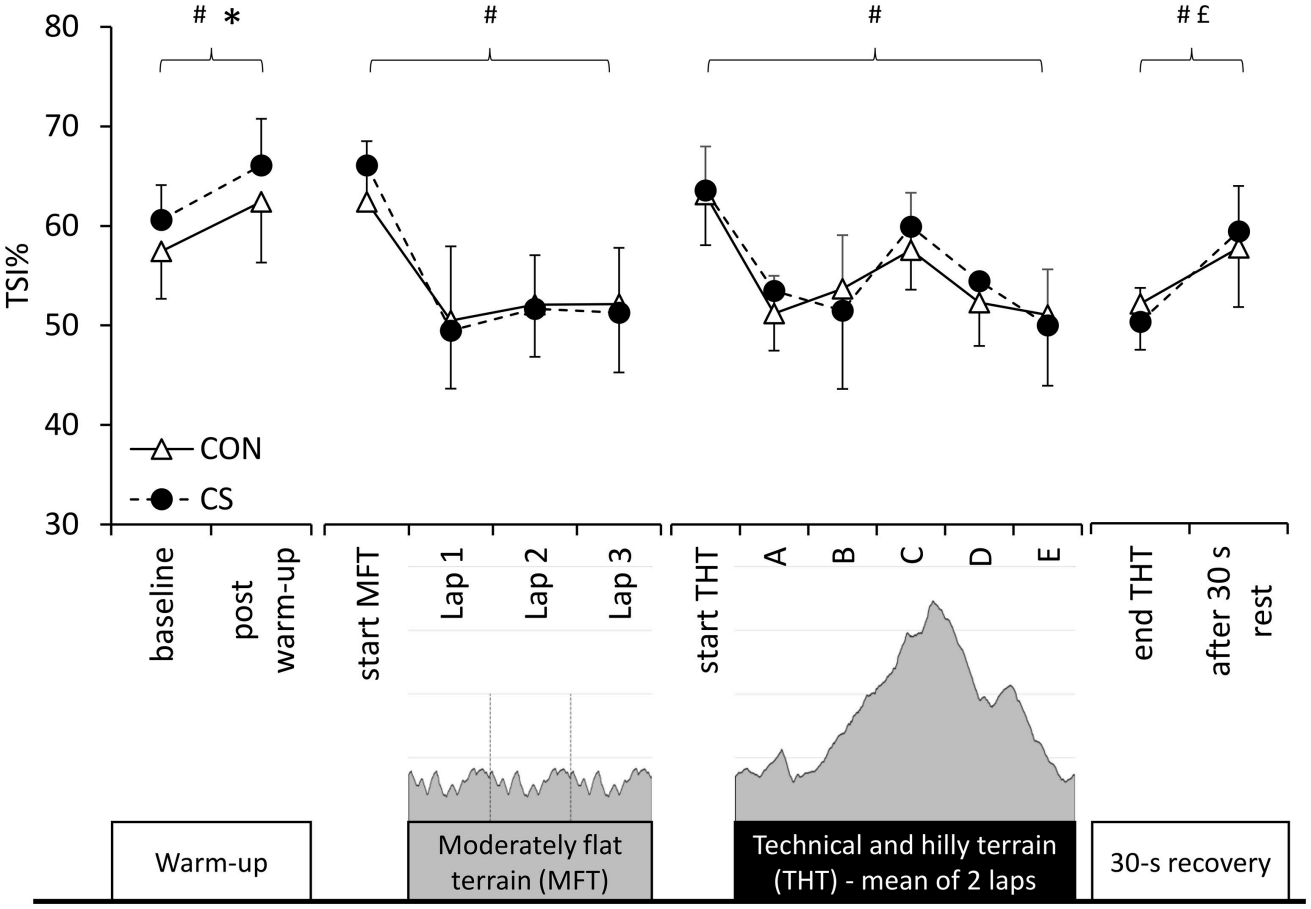
**Muscle oxygenation**. Muscle oxygenation (TSI) values at baseline and after warm-up, during running on moderately flat terrain (MFT) and technical and hilly terrain (THT), and directly after exercise wearing control (CON) or compression garments (CS). Symbols ^*^, #, and £ denote statistical significance at *p* < 0.05 for condition, time of measure and interaction, respectively. Data are mean ± SD.

Baseline measures performed prior to running revealed significant differences between conditions in local mBF (0.12 ± 0.05 vs. 0.17 ± 0.12 mL·min·100 mL^−1^ in CON and CS, respectively; 95% CI = −0.02, 0.09; *p* = 0.011; *d* = 0.54), but not in $\text{m}\dot{\text{V}}{\text{O}}_{2}$ (5.08 ± 2.07 vs. 5.35 ± 1.68 mL·min·100 g^−1^ in CON and CS, respectively; 95% CI = −0.26, 0.80; *p* = 0.280; *d* = 0.14). Significant differences were measured in TSI at baseline and after the warm-up for condition (*p* = 0.035, η^2^_*p*_ = 0.41) and time (*p* = 0.001, η^2^_*p*_ = 0.72) but not for interaction (*p* = 0.950, η^2^_*p*_ < 0.001; see Figure 2).

### MFT running bout

The average speed in each of the three laps was 10.4 ± 1.1 vs. 10.4 ± 1.1 km·h^−1^ in CON and CS, respectively. No significant effect of condition (*p* = 0.935, η^2^_*p*_ = 0.001), time (*p* = 0.681, η^2^_*p*_ = 0.03), or interaction (*p* = 0.125, η^2^_*p*_ = 0.15) were revealed. The average %HR in the three laps was 83.2 ± 5.7% and 83.3 ± 5.6% in CON and CS, respectively, and increased as a function of time (*p* = 0.008, η^2^_*p*_ = 0.31), but there were no significant effects of condition (*p* = 0.968, η^2^_*p*_ < 0.001) or interaction (*p* = 0.764, η^2^_*p*_ = 0.02).

There was a significant increase in mBF as a function of time (from 0.16 ± 0.05 to 0.34 ± 0.06 mL·min·100 mL^−1^ in CON and from 0.16 ± 0.05 to 0.28 ± 0.12 mL·min·100 mL^−1^ in CS; *p* < 0.001, η^2^_*p*_ = 0.89), but no effect of condition (*p* = 0.269, η^2^_*p*_ = 0.20) or interaction (*p* = 0.102, η^2^_*p*_ = 0.38). Likewise, $\text{m}\dot{\text{V}}{\text{O}}_{2}$ increased significantly (from 5.42 ± 2.11 to 9.66 ± 4.61 mL·min·100 g^−1^ in CON, and from 5.42 ± 1.78 to 9.36 ± 4.64 mL·min·100 g^−1^ in CS; *p* = 0.004, η^2^_*p*_ = 0.71), but no effect of condition (*p* = 0.817, η^2^_*p*_ = 0.008) or interaction (*p* = 0.814, η^2^_*p*_ = 0.009) was revealed. There was a significant effect of time (*p* < 0.001, η^2^_*p*_ = 0.89), but no effect of condition (*p* = 0.657, η^2^_*p*_ = 0.03) or interaction (*p* = 0.479, η^2^_*p*_ = 0.10) in TSI measured before, during and at the end of MFT (Figure 2).

**Figure 3:**
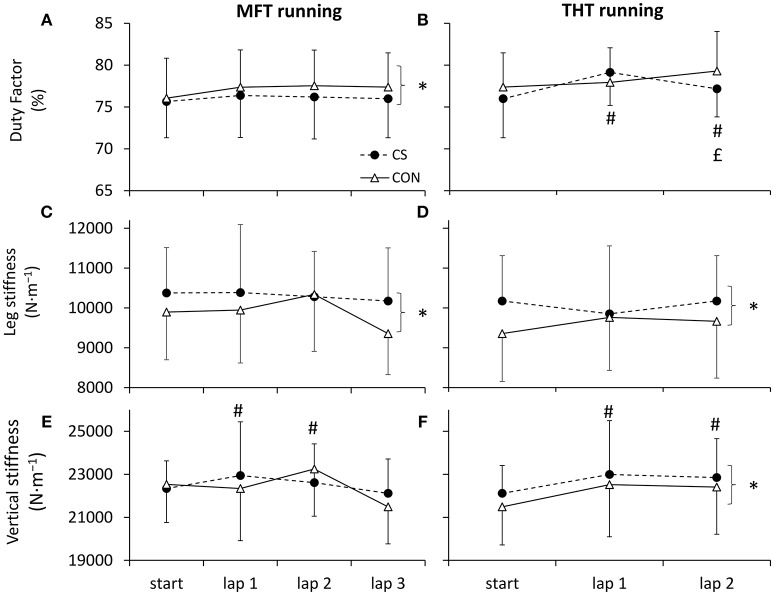
**Running pattern**. Variables of gait biomechanics duty factor (DF; **A,B**), leg stiffness (*k*_*leg*_; **C,D**) and vertical stiffness (*k*_*vert*_; **E,F**) during moderately flat (MFT) and technical and hilly (THT) running bouts wearing control (CON) or compression garments (CS). Symbols ^*^, #, and £ denote statistical significance at *p* < 0.05 for condition, time of measure and interaction, respectively. Data are mean ± SD.

Significant effects of condition were observed in running pattern variables with a greater *t*_*a*_, *k*_*leg*_, *F*_*max*_ and a smaller DF wearing CS compared to CON, and a significant decrease as a function of time was measured in *k*_*vert*_ and step frequency (Table 2 and Figure 3). Running speed in the optoelectric cells was constant across all tests (CON: 12.0 ± 0.6 km·h^−1^; CS: 11.9 ± 0.5 km·h^−1^) with no significant effect of condition (*p* = 0.281, η^2^_*p*_ = 0.09), time (*p* = 0.574, η^2^_*p*_ = 0.05) or interaction (*p* = 0.062, η^2^_*p*_ = 0.17).

**Table 2:** **Running biomechanics during MFT and THT**.

	**Time of measure**	**Mean ± *SD***			**Condition (CON vs. CS)**		**Time (lap)**		**Interaction (condition × lap)**	
		**CON**	**CS**		***p***	**η^2^*_*p*_***	***p***	**η^2^*_*p*_***	***p***	***η***^**2**^***_*****p*****_***
Contact time (ms)	PRE	0.222 ± 0.022	0.281 ± 0.019	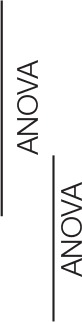	0.13	0.17	0.20	0.11	0.26	0.10
	MFT-lap 1	0.287 ± 0.023	0.281 ± 0.028							
	MFT-lap 2	0.282 ± 0.021	0.282 ± 0.024							
	MFT-lap 3	0.292 ± 0.020	0.284 ± 0.020							
	THT-lap 1	0.287 ± 0.022	0.287 ± 0.021		0.028^*^	**0.34**	0.98	0.00	0.17	0.14
	THT-lap 2	0.293 ± 0.020	0.282 ± 0.017							
Aerial time (ms)	PRE	0.089 ± 0.018	0.091 ± 0.017	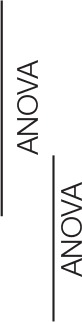	0.045^*^	**0.28**	0.18	0.12	0.69	0.04
	MFT-lap 1	0.084 ± 0.017	0.087 ± 0.018							
	MFT-lap 2	0.081 ± 0.015	0.088 ± 0.019							
	MFT-lap 3	0.082 ± 0.016	0.090 ± 0.018							
	THT-lap 1	0.081 ± 0.015	0.076 ± 0.014		0.32	0.08	0.002^*^	**0.39**	0.032^*^	**0.25**
	THT-lap 2	0.077 ± 0.018	0.084 ± 0.013							
Step frequency (Hz)	PRE	2.70 ± 0.13	2.69 ± 0.14	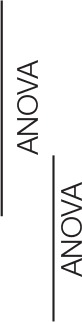	0.98	0.00	0.004^*^	**0.29**	0.08	0.16
	MFT-lap 1	2.70 ± 0.15	2.73 ± 0.18							
	MFT-lap 2	2.76 ± 0.11	2.71 ± 0.16							
	MFT-lap 3	2.65 ± 0.12	2.68 ± 0.12							
	THT-lap 1	2.72 ± 0.12	2.76 ± 0.13		0.09	0.23	<0.001^*^	**0.56**	0.97	0.00
	THT-lap 2	2.71 ± 0.11	2.74 ± 0.14							
Peak force (N)	PRE	1,470 ± 134	1,475 ± 127	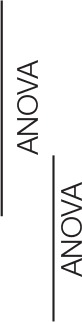	0.042^*^	**0.28**	0.39	0.07	0.77	0.03
	MFT-lap 1	1,444 ± 125	1,462 ± 131							
	MFT-lap 2	1,441 ± 136	1,465 ± 123							
	MFT-lap 3	1,444 ± 129	1,469 ± 128							
	THT-lap 1	1,432 ± 112	1,409 ± 115		0.18	0.14	0.016^*^	**0.29**	0.032^*^	**0.25**
	THT-lap 2	1,418 ± 111	1,446 ± 136							

*Inferential and effect size statistics for the variables of contact time, aerial time, peak force (F_max_) and step frequency before (PRE) and during the constant rate bout of running in control (CON) and compressive sleeves (CS) conditions. Bold values indicate statistically significant differences*.

### THT running bout

The average speed in each of the two laps was 8.9 ± 1.9 vs. 9.1 ± 1.9 km·h^−1^ in CON and CS, respectively. A significant effect of time (9.7 and 13.5% increase in lap 2 in CON and CS, respectively; *p* < 0.001, η^2^_*p*_ = 0.86), but not of condition (*p* = 0.149, η^2^_*p*_ = 0.17) or interaction (*p* = 0.633, η^2^_*p*_ = 0.02) were revealed. The average %HR was 85.7 ± 6.4% in CON and 87.6 ± 5.0% in CS and increased as a function of time (*p* = 0.014, η^2^_*p*_ = 0.41), but there were no effects of condition (*p* = 0.251, η^2^_*p*_ = 0.11) or interaction (*p* = 0.452, η^2^_*p*_ = 0.05).

The mBF measured before and after THT increased slightly but not significantly (from 0.28 ± 0.09 to 0.30 ± 0.08 mL·min·100mL^−1^ in CON, and from 0.28 ± 0.12 to 0.29 ± 0.15 mL·min·100 mL^−1^ in CS; *p* = 0.363, η^2^_*p*_ = 0.17) but there were no significant effects for condition (*p* = 0.869, η^2^_*p*_ = 0.01) or interaction (*p* = 0.822, η^2^_*p*_ = 0.01). Due to the inability of two subjects to tolerate high-level cuff inflation in a standing position after running and due to excessive noise during tests (no linear rate of increase/decrease in hemoglobin chromophores, as requested for a reliable estimation), there were insufficient complete data sets for $\text{m}\dot{\text{V}}{\text{O}}_{2}$ (*n* = 6), therefore those results are not reported. For TSI, there was a significant effect of time of measure within the laps (*p* < 0.001, η^2^_*p*_ = 0.78), but not of condition (*p* = 0.700, η^2^_*p*_ = 0.02), lap (*p* = 0.249, η^2^_*p*_ = 0.21) or interactions (condition × lap: *p* = 0.552, η^2^_*p*_ = 0.06; condition × time point: *p* = 0.504, η^2^_*p*_ = 0.13; lap × time of measure: *p* = 0.144, η^2^_*p*_ = 0.23; condition × lap × time of measure: *p* = 0.104, η^2^_*p*_ = 0.25). Since there was no effect of lap (lap 1 vs. lap 2), the average values of both laps are presented in Figure 2. Significant effects of time (*p* = 0.002, η^2^_*p*_ = 0.77) and interaction (*p* = 0.014, η^2^_*p*_ = 0.60), but no effect of condition (*p* = 0.970, η^2^_*p*_ < 0.001), were measured in the 30-s acute recovery phase directly following the end of exercise indicating a greater rate of recovery of TSI in CS compared to CON (Figure 2).

For the running pattern variables, a significant effect of condition was measured in *t*_*c*_, *k*_*leg*_, and *k*_*vert*_, a significant effect of time was measured in *t*_*a*_, *F*_*max*_, *k*_*vert*_ and step frequency, and an interaction was measured for *t*_*a*_ and *F*_*max*_ (Figure 3 and Table 2). Running speed in the optoelectric cells was constant across all tests (11.92 ± 0.54 vs. 11.88 ± 0.54 km·h^−1^ in CON and CS, respectively) with no significant effects of condition (*p* = 0.987, η^2^_*p*_ < 0.001), time (*p* = 0.151, η^2^_*p*_ = 0.16) or interaction (*p* = 0.402, η^2^_*p*_ = 0.06).

### Muscle functional capabilities

There were significant changes in all variables as a function of time (decreased average and maximum power, *t*_*c*_, and *k*_*leg*_, increased *t*_*a*_, and frequency), a significantly lower *t*_*c*_ in CS, as well as a tendency with large effect size for increased *k*_*leg*_ in CS, but no effect of condition or interaction (Table 3).

**Table 3:** **Muscle functional capabilities**.

	**Time of measure**	**Mean ± *SD***		**Condition (CON vs. CS)**		**Time (lap)**		**Interaction (condition × lap)**	
		**CON**	**CS**	***p***	**η^2^*_*p*_***	***p***	**η^2^*_*p*_***	***p***	**η^2^*_*p*_***
Average power (W·kg^−1^)	PRE	31.9 ± 5.85	31.7 ± 5.90	0.96	0.00	<0.001^*^	**0.73**	0.69	0.01
	POST	25.5 ± 5.51	25.9 ± 7.74						
Maximum power (W·kg^−1^)	PRE	37.3 ± 7.05	37.1 ± 6.13	0.86	0.00	<0.001^*^	**0.73**	0.94	0.00
	POST	31.0 ± 6.07	30.6 ± 7.97						
Contact time (ms)	PRE	0.196 ± 0.016	0.191 ± 0.014	0.034^*^	**0.30**	0.001^*^	**0.55**	0.41	0.05
	POST	0.206 ± 0.021	0.199 ± 0.018						
Aerial time (ms)	PRE	0.411 ± 0.047	0.405 ± 0.043	0.69	0.01	<0.001^*^	**0.64**	0.88	0.00
	POST	0.365 ± 0.049	0.360 ± 0.066						
Frequency (Hz)	PRE	1.65 ± 0.15	1.67 ± 0.13	0.44	0.05	0.003^*^	**0.51**	0.69	0.01
	POST	1.76 ± 0.18	1.80 ± 0.22						
Leg stiffness (N·m^−1^)	PRE	25.2 ± 2.48	26.2 ± 2.47	0.052^T^	0.26	0.035^*^	**0.31**	0.48	0.04
	POST	23.9 ± 3.41	25.4 ± 2.89						

*Inferential and effect size statistics for the variables of average and maximum power, contact time, aerial time, frequency and leg stiffness measured during the 20-s of maximal hopping performed before (PRE) and after (POST) running in control (CON) and compressive sleeves (CS) conditions. Bold values indicate statistically significant differences*.

#### Subjective sensations and DOMS

**Figure 4:**
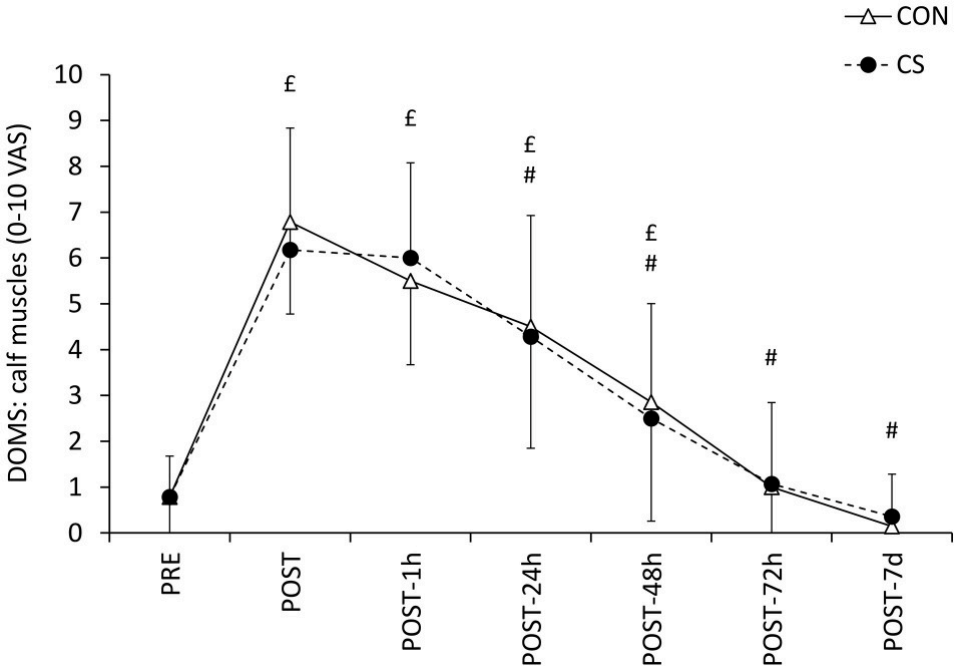
**Delayed Onset of Muscle Soreness**. Delayed Onset of Muscle Soreness (DOMS) in the calf muscles 1, 24, 48, 72 h, and 7 days after the running exercise performed wearing control (CON) or compression sleeves (CS). Symbols # and £ denote statistical significance at *p* < 0.05 compared to PRE and POST values, respectively. Data are mean ± SD.

The sensation of fatigue and pain increased PRE and POST exercise in calf and thigh muscles, and the increase was lower in the sensation of fatigue in thigh muscle in the CS condition (Table 4). The sensation of pain in the Achilles' tendon increased as a function of time, and this increase was significantly smaller in CS compared to CON (from 0.21 ± 0.58 to 2.93 ± 2.56 and from 0.21 ± 0.80 to 1.64 ± 1.91 in CON and CS, respectively; Wilcoxon signed-rank test: *Z* = 2.52, *p* = 0.012 [CS: PRE/POST]; *Z* = 2.80, *p* = 0.005 [CON: PRE/POST]; *Z* = 2.37, *p* = 0.018 [POST: CS/CON]). There was a significant increase in calf DOMS up to 48 h after the test (*p* < 0.001, η^2^_*p*_ = 0.79), but there were no effects of condition (*p* = 0.903, η^2^_*p*_ = 0.001) or interaction (*p* = 0.638, η^2^_*p*_ = 0.04) (Figure 4).

**Table 4:** **Subjective sensations of fatigue and pain**.

	**Time of measure**	**Mean** ±***SD***		**Condition (CON vs. CS)**		**Time (lap)**		**Interaction (condition × lap)**	
		**CON**	**CS**	***p***	**η^2^*_*p*_***	***p***	**η^2^*_*p*_***	***p***	**η^2^*_*p*_***
Calf muscle fatigue	PRE	0.79 ± 1.12	1.00 ± 0.96	0.44	0.05	<0.001^*^	**0.94**	0.13	0.17
	POST	7.21 ± 0.97	6.46 ± 1.89						
Thigh muscle fatigue	PRE	1.29 ± 0.99	0.86 ± 0.86	0.041^*^	**0.28**	<0.001^*^	**0.95**	0.53	0.00
	POST	6.71 ± 1.44	5.93 ± 3.00						
Calf muscle pain	PRE	0.79 ± 1.19	0.79 ± 0.89	0.54	0.03	<0.001^*^	**0.90**	0.55	0.03
	POST	6.79 ± 2.01	6.18 ± 2.66						
Thigh muscle pain	PRE	0.57 ± 0.76	0.64 ± 0.84	0.15	0.15	<0.001^*^	**0.88**	0.10	0.19
	POST	6.21 ± 2.33	5.29 ± 2.30						

*Inferential and effect size statistics for the variables of perceived calf muscle fatigue, thigh muscle fatigue, calf muscle pain and thigh muscle pain measured using 0–10 visual analog scales before (PRE) and after (POST) running in control (CON) and compressive sleeves (CS) conditions. Bold values indicate statistically significant differences*.

## Discussion

This randomized, cross-over controlled study aimed to measure the effects of wearing calf compression sleeves compared to control garments during prolonged trail running on physiological and biomechanical parameters, exercise performance, and subjective perception of muscle fatigue, pain and soreness. The main findings of this study were that wearing CS compared to CON during trail running (i) improved muscle oxygenation before and after exercise (in a stationary position), but did not differentially affect local muscle oxygenation or heart rate during running on moderately flat or technical and hilly terrain, including during sustained uphill or downhill running, (ii) modified running pattern and muscle mechanical capabilities during hopping via increased leg stiffness, (iii) improved the perception of pain in the Achilles' tendon, and (iv) did not affect performance in all-out trail running with marked elevation gain and loss, which confirms findings of previous studies in shorter duration exercise (Vercruyssen et al., 2012, 2016; Bieuzen et al., 2014).

As expected, HR increased as a function of time (cardiac drift) but we did not find any difference across garment conditions. This result is similar to those of previous studies during (Ali et al., 2007; Duffield and Portus, 2007; Sperlich et al., 2011; Wahl et al., 2011; Vercruyssen et al., 2012; Born et al., 2014; Priego Quesada et al., 2015) or after exercise (Duffield et al., 2010; Ménétrier et al., 2011). On the contrary, a beneficial effect of wearing CS was found in muscle oxygenation during all resting situations, including after the test, which was likely due to an increased perfusion and reduced venous pooling in CS, as previously shown (Bochmann et al., 2005; Bringard et al., 2006; Ménétrier et al., 2011). Changes in skin temperature and skin blood flow have also been pointed to explain part of the improved tissue saturation previously reported with the application of external compression, but these mechanisms are unlikely to play a prominent role in the current study as the control condition we used induced similar calf skin temperature during running compared to CS (cf. Table 1). This result is also in agreement with previous studies having reported improvements in muscle oxygenation before and after running (Bringard et al., 2006; Sear et al., 2010; Ménétrier et al., 2011) and cycling exercises (Scanlan et al., 2008), as we also measured a beneficial effect of wearing CS in all resting situations (muscle oxygenation improved after donning the CS, after warming up, and after the test). However, our study is the first to report findings of muscle oxygenation during prolonged trail running, for which we measured no effect of exercise duration (fatigue) or condition (compression), including during sustained uphill and downhill running. Therefore, it is likely that the pressure in the muscular compartment during running exercises performed on various gradients exceeds the pressure exerted by CS, which could blunt its potential beneficial effects. Previous studies have indicated that increasing the mechanical pressure exerted by compression garments to approximately 40 mmHg had no effects during running at submaximal and maximal intensities in laboratory conditions (Wahl et al., 2011) and could even have detrimental effects on blood flow during cycling exercise (Sperlich et al., 2013). However, by the same mechanism, CS could potentially have ergogenic effects during repeated exercise/recovery phases where the recovery rate in muscle oxygenation is beneficial, such as intermittent exercise (Sear et al., 2010), but also possibly for exercise performed on multiple days. The two sessions were conducted in similar conditions, as no statistical differences were observed in factors that may impact parameters of interest (i.e., ambient and skin temperatures, perceived fatigue and pain before the test, the time separating MFT and THT bouts, and the time to complete the MHT bout).

For the first time, changes in running biomechanics induced by CS were reported during exercise. Beyond the effects of exercise likely imputable to fatigue (large effect sizes for increased step frequency, decreased aerial time, significant increases in vertical stiffness and duty factor), we reported (i) a lower duty factor and greater leg stiffness during the MFT running bout in CS compared to CON (influenced mostly by increases in aerial time), and (ii) greater leg stiffness and vertical stiffness, as well as an interaction effect in duty factor during THT running in CS compared to CON (mostly influenced by decreases in contact time). For lower limb functional mechanical capabilities, while all variables were affected by exercise, we also reported a decrease in contact time and a large tendency for increases in leg stiffness with CS. Previous studies have indicated that the biomechanical adaptations induced by prolonged running were associated with similar trends, such as higher step frequency, smaller ground reaction forces contributing to increased leg and vertical stiffness after a 24-h treadmill run (Morin et al., 2011b), reduced aerial time and increased step frequency contributing to a greater vertical stiffness after a 166-km mountain ultramarathon (Morin et al., 2011a), or higher step frequency, duty factor and lower aerial time and ground reaction force after 8,500 km of running in 161 days (Millet et al., 2009). These biomechanical adaptations were suggested to be related to a smoother and safer running pattern preventing the impact of additional important mechanical constraints on the musculo-skeletal system. In the current study, a higher leg stiffness was also observed with CS compared to CON before, during and after running on level and hilly terrain, and in maximal hopping. However, in contrast with the above-mentioned previous findings associating the increase in leg stiffness with an increase in DF, the higher leg stiffness observed in the current study was associated with lower DF, either via a longer *t*_*a*_ and higher peak vertical force during MFT, or a shorter *t*_*c*_ during HFT and hopping. Therefore, the acute increases in leg stiffness associated with a lower relative time spent in contact with the ground can be interpreted as a more dynamical running pattern and leg mechanical behavior. Previous studies have suggested such biomechanical improvements may reside in the ergogenic aid of compression garments in reducing muscle oscillations (Kraemer et al., 1998; Mizuno et al., 2016), potentially through enhanced proprioceptive capability (Kraemer et al., 1998). However, there is currently no systematic study of the mechanisms underlying these acute changes in leg mechanical behavior wearing CS, and further investigations are required.

In this study, we reported that wearing CS resulted in a lower perception of fatigue in thigh muscles, and for the first time, a lower pain in the Achilles' tendon immediately after exercise. We also observed no effect of compression effect in DOMS up to 7 days after the test, which extends the findings of several other studies (Ali et al., 2011; Bovenschen et al., 2013; Driller and Halson, 2013; Areces et al., 2015; Chan et al., 2016). However, this result differs to that of Bieuzen et al. (2014), where the DOMS measured 1-h and 24-h POST trail running were likely to be lower in the compression condition. The differential alteration in the perceived sensation of fatigue, pain and soreness at the different muscle and tendon sites cannot be elucidated with the current results. Future investigations are needed to precise these discrepancies and may explore the potential beneficial implications of such sleeves, especially when used chronically, since CS could provide analgesic or prophylactic effects by enhancing proprioception and/or reducing the transmission of potentially harmful vibrations (Macefield, 2005). However, despite the improvements in the perception of pain in the Achilles' tendon, and despite the absence of deleterious effects on any other variable, we did not measure improvements in exercise performance in either running (measured using time to complete the large laps) or muscle mechanical capabilities (hopping power). Therefore, it is possible to formulate the hypothesis that (i) the exercise used in the current study may have not been sufficient in duration and/or did not generate sufficient neuromuscular alterations in order for the potential protective action of CS to be transferred in exercise performance (as evaluated from this experimental design), or that (ii) the control garments provided some level of haptic feedback despite the absence of compression, akin to a placebo effect. Finally, the current study was performed in a cohort of trained trail runners and therefore, the potential protective effect of CS during trail running may have been minimized compared to a cohort of participants unfamiliar with running, or unfamiliar with running in terrain with marked elevation gain and loss.

## Conclusion

This study shows for the first time that wearing calf compression sleeves during prolonged (~2 h 30 min) trail running modified running biomechanics and lower limb muscle functional capabilities toward a more dynamic behavior, and reduced perception of pain in Achilles' tendon compared to a control condition without compression. In line with previous studies performed in traditional on-road running, or in shorter duration trail running, wearing calf compression sleeves did not modify exercise performance, muscle oxygenation and heart rate. Future studies are required to investigate why the selective improvement of stride biomechanics and Achilles' tendon pain had no effect on exercise performance, using either longer exercise duration or running performed in even more challenging terrain. Wearing compression sleeves during running appears to be associated with no adverse effects on the measured variables.

## Author contributions

Conceived and designed the experiment: TR, PS, MaPa, and GM. Performed the experiment: TR, MaPi, and FD. Analyzed the data: HK, TR, PS, and MaPi. Wrote the paper: HK, TR, PS, GM, MaPa, and FD.

## Funding

This study (REC'UP study) was supported using specific funds and equipment from compression garment manufacturer Thuasne (France).

### Conflict of interest statement

The authors declare that the research was conducted in the absence of any commercial or financial relationships that could be construed as a potential conflict of interest.

## References

[B1] Ali A. Caine M. P. Snow B. (2007). Graduated compression stockings: physiological and perceptual responses during and after exercise. J. Sports Sci. 25, 413–419. 10.1080/02640410600718376 17365528

[B2] Ali A. Creasy R. Edge J. H. (2011). The effect of graduated compression stockings on running performance. J. Strength Condition. Res. 25, 1385–1392. 10.1519/JSC.0b013e3181d6848e 21293307

[B3] Ali A. Creasy R. H. Edge J. H. (2010). Physiological effects of wearing graduated compression stockings during running. Eur. J. Appl. Physiol. 109, 1017–1025. 10.1007/s00421-010-1447-1 20354717

[B4] Areces F. Salinero J. J. Abian-Vicen J. González-Millán C. Ruiz-Vicente D. Lara B.. (2015). The use of compression stockings during a marathon competition to reduce exercise-induced muscle damage: are they really useful? J. Orthopaed. Sports Phys. Ther. 45, 462–470. 10.2519/jospt.2015.5863 25899215

[B5] Bieuzen F. Brisswalter J. Easthope C. Vercruyssen F. Bernard T. Hausswirth C. (2014). Effect of wearing compression stockings on recovery after mild exercise-induced muscle damage. Int. J. Sports Physiol. Perform. 9, 256–264. 10.1123/ijspp.2013-0126 23751727

[B6] Bochmann R. P. Seibel W. Haase E. Hietschold V. Rödel H. Deussen A. (2005). External compression increases forearm perfusion. J. Appl. Physiol. 99, 2337–2344. 10.1152/japplphysiol.00965.2004 16081618

[B7] Born D.-P. Holmberg H.-C. Goernert F. Sperlich B. (2014). A novel compression garment with adhesive silicone stripes improves repeated sprint performance – a multi-experimental approach on the underlying mechanisms. BMC Sports Sci. Med. Rehabil. 6:21. 10.1186/2052-1847-6-21 24914412 PMC4049427

[B8] Born D.-P. Sperlich B. Holmberg H.-C. (2013). Bringing light into the dark: effects of compression clothing on performance and recovery. Int. J. Sports Physiol. Perform. 8, 4–18. 10.1123/ijspp.8.1.4 23302134

[B9] Bosco C. Luhtanen P. Komi P. (1983). A simple method for measurement of mechanical power in jumping. Eur. J. Appl. Physiol. 50, 273–282. 10.1007/BF00422166 6681758

[B10] Bovenschen H. J. Te Booij M. Van Der Vleuten C. J. M. (2013). Graduated compression stockings for runners: friend, foe, or fake? J. Athl. Train. 48, 226–232. 10.4085/1062-6050-48.1.26 23672387 PMC3600925

[B11] Bringard A. Denis R. Belluye N. Perrey S. (2006). Effects of compression tights on calf muscle oxygenation and venous pooling during quiet resting in supine and standing positions. J. Sports Med. Fitness 46, 548–554. 17119519

[B12] Bullimore S. R. Burn J. F. (2006). Consequences of forward translation of the point of force application for the mechanics of running. J. Theor. Biol. 238, 211–219. 10.1016/j.jtbi.2005.05.011 15996682

[B13] Chan V. Duffield R. Watsford (2016). The effects of compression garments on performance of prolonged manual-labour exercise and recovery. Appl. Physiol. Nutr. Metab. 41, 125–132. 10.1139/apnm-2015-0335 26778138

[B14] Dalleau G. Belli A. Lacour J.-R. Bourdin M. (2004). A simple method for field measurements of leg stiffness in hopping. Int. J. Sports Med. 25, 170–176. 10.1055/s-2003-45252 15088239

[B15] Di Prampero P. E. Boutellier U. Pietsch P. (1983). Oxygen deficit and stores at onset of muscular exercise in humans. J. Appl. Physiol. 55, 146–153. 6885564 10.1152/jappl.1983.55.1.146

[B16] Doan K. Kwon Y.-H. Newton R. U. Shim J. Popper E. M. Rogers R. A.. (2003). Evaluation of a lower-body compression garment. J. Sports Sci. 21, 601–610. 10.1080/0264041031000101971 12875311

[B17] Driller M. W. Halson S. L. (2013). The effects of lower-body compression garments on recovery between exercise bouts in highly-trained cyclists. J. Sci. Cycling 2, 45–50.

[B18] Duffield R. Cannon J. King M. (2010). The effects of compression garments on recovery of muscle performance following high-intensity sprint and plyometric exercise. J. Sci. Med. Sport 13, 136–140. 10.1016/j.jsams.2008.10.006 19131276

[B19] Duffield R. Edge J. Merrells R. Hawke E. Barnes M. Simcock D.. (2008). The effects of compression garments on intermittent exercise performance and recovery on consecutive days. Int. J. Sports Physiol. Perform. 3, 454–468. 10.1123/ijspp.3.4.454 19223671

[B20] Duffield R. Portus M. (2007). Comparison of three types of full-body compression garments on throwing and repeat-sprint performance in cricket players. Br. J. Sport Med. 41, 409–414. 10.1136/bjsm.2006.033753 17341589 PMC2465357

[B21] Duncan A. Meek J. H. Clemence M. Elwell C. E. Fallon P. Tyszczuk L.. (1996). Measurement of cranial optical path length as a function of age using phase resolved Near Infrared Spectroscopy. Pediatr. Res. 39, 889–894. 10.1203/00006450-199605000-00025 8726247

[B22] Engel F. A. Holmberg H.-C. Sperlich B. (2016). Is there evidence that runners can benefit from wearing compression clothing? Sports Med. 46, 1–14. 10.1007/s40279-016-0546-5 27106555

[B23] Giandolini M. Gimenez P. Temesi J. Arnal P. J. Martin V. Rupp T.. (2016). Effect of the fatigue Induced by a 110-km ultramarathon on tibial impact acceleration and lower leg kinematics. PLoS ONE 11:e0151687. 10.1371/journal.pone.0151687 27031830 PMC4816299

[B24] Hamaoka T. Mccully K. K. Quaresima V. Yamamoto K. Chance B. (2007). Near-infrared spectroscopy/imaging for monitoring muscle oxygenation and oxidative metabolism in healthy and diseased humans. J. Biomed. Opt. 12:062105. 10.1117/1.2805437 18163808

[B25] Hawker G. A. Mian S. Kendzerska T. French M. (2011). Measures of adult pain. Arthritis Care Res. 63, 240–252. 10.1002/acr.20543 22588748

[B26] Higgins T. Naughton G. A. Burgess D. (2009). Effects of wearing compression garments on physiological and performance measures in a simulated game-specific circuit for netball. J. Sci. Med. in Sport 12, 223–226. 10.1016/j.jsams.2007.08.018 18078789

[B27] Hill J. Howatson G. Van Someren K. Leeder J. Pedlar C. (2014). Compression garments and recovery from exercise-induced muscle damage: a meta-analysis. Br. J. Sport Med. 48, 1340–1346. 10.1136/bjsports-2013-092456 23757486

[B28] Joseph C. W. Bradshaw E. J. Kemp J. Clark R. A. (2013). The interday reliability of ankle, knee, leg, and vertical musculoskeletal stiffness during hopping and overground running. J. Appl. Biomech. 29, 386–394. 10.1123/jab.29.4.386 22923423

[B29] Kemmler W. V. S. Simon Köckritz, C. Mayhew J. Wassermann A. Zapf J. (2009). Effect of compression stockings on running performance in men runners. J. Strength Condit. Res. 23, 101–105. 10.1519/JSC.0b013e31818eaef3 19057400

[B30] Kerhervé H. A. Millet G. Y. Solomon C. (2015). The dynamics of speed selection and psycho-physiological load during a mountain ultramarathon. PLoS ONE 10:e0145482. 10.1371/journal.pone.0145482 26691599 PMC4687124

[B31] Kraemer W. J. Bush J. A. Newton R. U. Duncan N. D. Volek J. S. Denegar C. R. (1998). Influence of a compression garment on repetitive power output production before and after different types of muscle fatigue. Sports Med. Train. Rehabil. 8, 163–184. 10.1080/15438629809512525

[B32] Lee K. A. Hicks G. Nino-Murcia G. (1991). Validity and reliability of a scale to assess fatigue. Psychiatry Res. 36, 291–298. 10.1016/0165-1781(91)90027-M 2062970

[B33] Macefield V. G. (2005). Physiological characteristics of low-threshold mechanoreceptors in joints, muscle and skin in human subjects. Clin. Exp. Pharmacol. Physiol. 32, 135–144. 10.1111/j.1440-1681.2005.04143.x 15730450

[B34] MacRae B. A. Cotter J. D. Laing R. M. (2011). Compression garments and exercise: garment considerations, physiology and performance. Sports Med. 41, 815–843. 10.2165/11591420-000000000-00000 21923201

[B35] Ménétrier A. Mourot L. Bouhaddi M. Regnard J. Tordi N. (2011). Compression sleeves increase tissue oxygen saturation but not running performance. Int. J. Sports Med. 32, 864–868. 10.1055/s-0031-1283181 22052027

[B36] Millet G. Y. (2011). Can neuromuscular fatigue explain running strategies and performance in ultra-marathons? The flush model. Sports Med. 41, 489–506. 10.2165/11588760-000000000-00000 21615190

[B37] Millet G. Y. Morin J.-B. Degache F. Edouard P. Féasson L. Verney J.. (2009). Running from Paris to Beijing: biomechanical and physiological consequences. Eur. J. Appl. Physiol. 107, 731–738. 10.1007/s00421-009-1194-3 19756701

[B38] Miyamoto N. Hirata K. Mitsukawa N. Yanai T. Kawakami Y. (2011). Effect of pressure intensity of graduated elastic compression stocking on muscle fatigue following calf-raise exercise. J. Elect. Kinesiol. 21, 249–254. 10.1016/j.jelekin.2010.08.006 20843703

[B39] Mizuno S. Morii I. Tsuchiya Y. Goto K. (2016). Wearing compression garment after endurance exercise promotes recovery of exercise performance. Int. J. Sports Med. 37, 870–877. 10.1055/s-0042-106301 27454135

[B40] Morin J.-B. Samozino P. Millet G. Y. (2011a). Changes in running kinematics, kinetics, and spring-mass behavior over a 24-h run. Med. Sci. Sports Exerc. 43, 829–836. 10.1249/MSS.0b013e3181fec518 20962690

[B41] Morin J.-B. Tomazin K. Edouard P. Millet G. Y. (2011b). Changes in running mechanics and spring–mass behavior induced by a mountain ultra-marathon race. J. Biomech. 44, 1104–1107. 10.1016/j.jbiomech.2011.01.028 21342691

[B42] Partsch H. Flour M. Coleridge-Smith P. International Compression, Club Uhl J.-F. (2008). Indications for compression therapy in venous and lymphatic disease consensus based on experimental data and scientific evidence. Under the auspices of the IUP. Int. Angiol. 27, 193–219. 18506124

[B43] Priego Quesada J. I. Lucas-Cuevas Á. G. Aparicio I. Giménez J. V. Cortell-Tormo J. M. Pérez-Soriano P. (2015). Long-term effects of graduated compression stockings on cardiorespiratory performance. Biol. Sport 32, 219–223. 10.5604/20831862.1150304 26424925 PMC4577560

[B44] Rugg S. Sternlicht E. (2013). The effect of graduated compression tights, compared with running shorts, on counter movement jump performance before and after submaximal running. J. Strength Condit. Res. 27, 1067–1073. 10.1519/JSC.0b013e3182610956 22692109

[B45] Scanlan A. T. Dascombe B. Reaburn P. R. Osborne M. (2008). The effects of wearing lower-body compression garments during endurance cycling. Int. J. Sports Physiol. Perform. 3, 424–438. 10.1123/ijspp.3.4.424 19223669

[B46] Sear J. A. Hoare T. K. Scanlan A. T. Abt G. A. Dascombe B. (2010). The effects of whole-body compression garments on prolonged high-intensity intermittent exercise. J. Strength Condit. Res. 24, 1901–1910. 10.1519/JSC.0b013e3181db251b 20555284

[B47] Sperlich B. Born D.-P. Kaskinoro K. Kalliokoski K. K. Laaksonen M. S. (2013). Squeezing the muscle: compression clothing and muscle metabolism during recovery from high intensity exercise. PLoS ONE 8:e60923. 10.1371/journal.pone.0060923 23613756 PMC3629206

[B48] Sperlich B. Haegele M. Krüger M. Schiffer T. Holmberg H.-C. Mester J. (2011). Cardio-respiratory and metabolic responses to different levels of compression during submaximal exercise. Phlebology 26, 102–106. 10.1258/phleb.2010.010017 21228356

[B49] Stickford A. S. L. Chapman R. F. Johnston J. D. Stager J. M. (2015). Lower-leg compression, running mechanics, and economy in trained distance runners. Int. J. Sports Physiol. Perform. 10, 76–83. 10.1123/ijspp.2014-0003 24911991

[B50] Tanaka H. Monahan K. D. Seal D. R. (2001). Age-predicted maximal heart rate revisited. J. Am. College Cardiol. 1, 153–156. 10.1016/S0735-1097(00)01054-8 11153730

[B51] Valle X. Til L. Drobnic F. Turmo A. Montoro J. B. Valero O.. (2013). Compression garments to prevent delayed onset muscle soreness in soccer players. Muscles Ligaments Tendons J. 3, 295–302. 24596693 PMC3940503

[B52] Van Beekvelt M. C. P. (2002). Quantitative Near-Infrared Spectroscopy in Human Skeletal Muscle. Methodological Issues and Clinical Application. Ph.D. University of Nijmegen.

[B53] Varela-Sanz A. España J. Carr N. Boullosa D. A. Esteve-Lanao J. (2011). Effects of gradual-elastic compression stockings on running economy, kinematics, and performance in runners. J. Strength Condit. Res. 25, 2902–2910. 10.1519/JSC.0b013e31820f5049 21912341

[B54] Vercruyssen F. Easthope C. Bernard T. Hausswirth C. Bieuzen F. Gruet M.. (2012). The influence of wearing compression stockings on performance indicators and physiological responses following a prolonged trail running exercise. Eur. J. Sport Sci. 14, 144–150. 10.1080/17461391.2012.730062 24533521

[B55] Vercruyssen F. Gruet M. Colson S. Ehrstrom S. Brisswalter J. (2016). Compression garments, muscle contractile function and economy in trail runners. Int. J. Sports Physiol. Perform. 12, 62–68. 10.1123/ijspp.2016-0035 27081007

[B56] Vernillo G. Savoldelli A. Zignoli A. Trabucchi P. Pellegrini B. Millet G. P.. (2014). Influence of the world's most challenging mountain ultra-marathon on energy cost and running mechanics. Eur. J. Appl. Physiol. 114, 929–939. 10.1007/s00421-014-2824-y 24477570

[B57] Wahl P. Bloch W. Mester J. Born D.-P. Sperlich B. (2011). Effects of different levels of compression during sub-maximal and high-intensity exercise on erythrocyte deformability. Eur. J. Appl. Physiol. 112, 2163–2169. 10.1007/s00421-011-2186-7 21964909

